# IL-6 and IGF-1 Signaling Within and Between Muscle and Bone: How Important is the mTOR Pathway for Bone Metabolism?

**DOI:** 10.1007/s11914-015-0264-1

**Published:** 2015-02-25

**Authors:** Astrid D. Bakker, Richard T. Jaspers

**Affiliations:** 1 Laboratory for Myology, MOVE Research Institute Amsterdam, Faculty of Human Movement Sciences, VU University Amsterdam, Van der Boechorststraat 9, 1081 BT Amsterdam, The Netherlands; 2 Department of Oral Cell Biology, Academic Centre for Dentistry Amsterdam (ACTA), University of Amsterdam and VU University Amsterdam, MOVE Research Institute Amsterdam, Gustav Mahlerlaan 3004, 1081 LA Amsterdam, The Netherlands

**Keywords:** Osteocyte, Osteoblast, Myoblast, Hypertrophy, mTOR, Mechanical loading, Aging, Osteoporosis

## Abstract

Insulin-like growth factor 1 (IGF-1) and interleukin 6 (IL-6) play an important role in the adaptation of both muscle and bone to mechanical stimuli. Here, we provide an overview of the functions of IL-6 and IGF-1 in bone and muscle metabolism, and the intracellular signaling pathways that are well known to mediate these functions. In particular, we discuss the Akt/mammalian target of rapamycin (mTOR) pathway which in skeletal muscle is known for its key role in regulating the rate of mRNA translation (protein synthesis). Since the role of the mTOR pathway in bone is explored to a much lesser extent, we discuss what is known about this pathway in bone and the potential role of this pathway in bone remodeling. We will also discuss the possible ways of influencing IGF-1 or IL-6 signaling by osteocytes and the clinical implications of pharmacological or nutritional modulation of the Akt/mTOR pathway.

## Introduction

Most people know from personal experience that muscles increase in mass when loaded and decrease in mass during a period of physical inactivity. Much less commonly known is that exactly the same principle holds true for bones. This adaptation of bone mass to mechanical demands is facilitated by the activity of osteoblasts and osteoclasts. Osteoclasts remove bone in places of relative unloading, while osteoblasts form bone in places of relative high mechanical loading, leading to a constant adaptation of bone mass and structure [1]. As a result, healthy bones are able to withstand the forces placed upon them, while using a minimum of material. During osteoporosis, this delicate balance between bones’ resistance against mechanical loads and bone mass is clearly disturbed, mostly as a result of enhanced bone loss [2]. Although most studies have focused on the role of osteoblasts and osteoclasts in the emergence of osteoporosis, osteocytes may well be involved in the pathogenesis of this disease. It is well known that osteocytes produce signaling molecules in response to mechanical loading which affect osteoclast and osteoblast recruitment and activity [3, 4]. Our group has shown that nitric oxide and prostaglandin production in response to mechanical loading of cultured osteocytes from osteoporotic individuals differs from that of osteocytes derived from people with a high bone mass [5]. As such, alterations in the osteocyte responses to mechanical loading, in terms of signaling molecule production, may well be involved in the etiology of osteoporosis.

In recent years, a defined picture is emerging of osteocytes as signaling centers, actively communicating with osteoblasts, osteoclasts, marrow cells, cells of the lymphatic system, and the kidney. In addition, potential lines of communication between bone and muscle are currently under much scrutiny [6–9]. The organ most closely connected to the skeleton is arguably the musculature. If bones indeed communicate with muscle, the most likely source of the paracrine and endocrine signal is the osteocyte, which is by far the most abundant cell in adult bones, and osteocyte-derived signals have been shown to reach remote organs [10]. Presuming that osteocyte communication with muscles affects muscle mass, the opposite may also be true. Muscles may communicate to osteocytes, or muscles could communicate directly with osteoblasts and osteoclasts via similar signals that osteocytes employ to dictate osteoclast and osteoblast behavior. Osteocytes and muscle cells show considerable overlap in the molecules they use to regulate their mass in response to mechanical cues. Our group recently showed that differentiated myotubes and osteocytes alike cells produce factors such as nitric oxide (NO), hepatocyte growth factor (HGF), vascular endothelial growth factor (VEGF), insulin-like growth factor 1 (IGF-1), and interleukin 6 (IL-6) in response to mechanical loading [11•, 12]. Short-lived molecules are unlikely to do more than autocrine and some paracrine signaling, but elevated levels of muscle-derived IL-6 and IGF-1 are found in the circulation after vigorous exercise [13–15]. We have shown that IL-6 derived from myotubes affects formation of osteoclasts in vitro [12]. This indicates that communication between muscle and bone is theoretically possible. Whether muscle and bone indeed communicate via the production of soluble factors is difficult to establish, but some evidence is available in support of this hypothesis [6, 16]. One complicating factor is that muscles exert mechanical forces in bone, thereby affecting bone mass regardless of the existence of an exchange of biochemical factors. Considering this, it is possible that osteoporosis is simply related to a decrease in the number and/or magnitude of mechanical stimuli in bone, caused by the loss of muscle mass that is common with aging. Maintenance of muscle mass could thus be a therapeutic option in elderly, in order to preserve bone integrity and mass. Even if it turns out that biochemical communication between muscle and bone does not play a significant role in bone homeostasis, it is likely that, considering the similarities in signaling molecules employed by both tissue types, much can be learned from the muscle field in order to generate a better understanding of bone mass regulation and vice versa.

## Insulin-Like Growth Factor 1

IGF-1 is a hormone rather similar in molecular structure to insulin. It is produced primarily by the liver under the control of growth hormone, plays an important role in regulating growth in children, and has anabolic effects in adults [17]. By far, the largest amount of IGF-1 in the body is bound to IGF-binding proteins (IGFBPs), which affect the activity of IGF-1. For instance, IGFBP-2 and IGFBP-5 bind IGF-1 at a higher affinity than its receptor, and an increased serum level of these IGFBPs thus result in lower IGF-1 signaling [18]. Several splice variants from the IGF-1 gene have been identified, of which IGF-1Ea and IGF-1Eb/c (also known as mechano growth factor (MGF)) play a role in muscle and bone homeostasis. These two splice variants differ with respect to their nucleotide sequence in exon 5 which encompasses an insert of about 23 amino acids, coding for the E-peptide [19]. The 70 kD IGF-1 domain, coded by exons 3 and 4 of the IGF-1 gene, stimulates osteocyte and osteoblast survival [20, 21], as well as osteoblast differentiation and matrix production [22], while the E peptide in MGF promotes MC3T3-E1 osteoblast proliferation [23]. In addition to these anabolic effects in bone, the 70 kD IGF-1 domain has been shown to promote osteoclastogenesis [24, 25].

Besides being an endocrine molecule, IGF-1 also exerts effects in a paracrine/autocrine manner in many tissues. IGF-1 is produced locally in bone and is able to exert effects in bone in an autocrine manner [26]. Since then, the question “how much of the anabolic effect of IGF-1 in bone is autocrine in nature?” has remained unsolved until Elis et al. showed that, in the absence of tissue-specific IGF-1 gene expression, maintaining long-term elevated IGF-1 levels in serum by IGF-1 gene overexpression in the liver was sufficient to restore skeletal architecture and mechanical function [27•]. In addition, conditional disruption of the IGF-1 gene in osteocytes under the control of the DMP-1 promoter slightly reduces bone mineral content, but not mineral density, and IGF-1 knockout in osteocytes does not alter plasma levels of IGF-1 [28]. This suggests that IGF-1 produced locally in bone has a minor role in bone development compared to IGF-1 produced in the liver. However, the importance of local production of IGF-1 in bone in response to mechanical loading may be a different story altogether. Mechanical loading is anabolic for bone, and osteocytes have been shown to produce IGF-1 in response to mechanical loading in vitro and in vivo [29•, 30]. In addition, mechanical loading increases bone formation in wild-type mice but not in mice with osteocytes deficient in IGF-1 [31••]. From the above, it was concluded that IGF-1 plays a pivotal role in the response of bone to mechanical forces.

**Fig. 1:**
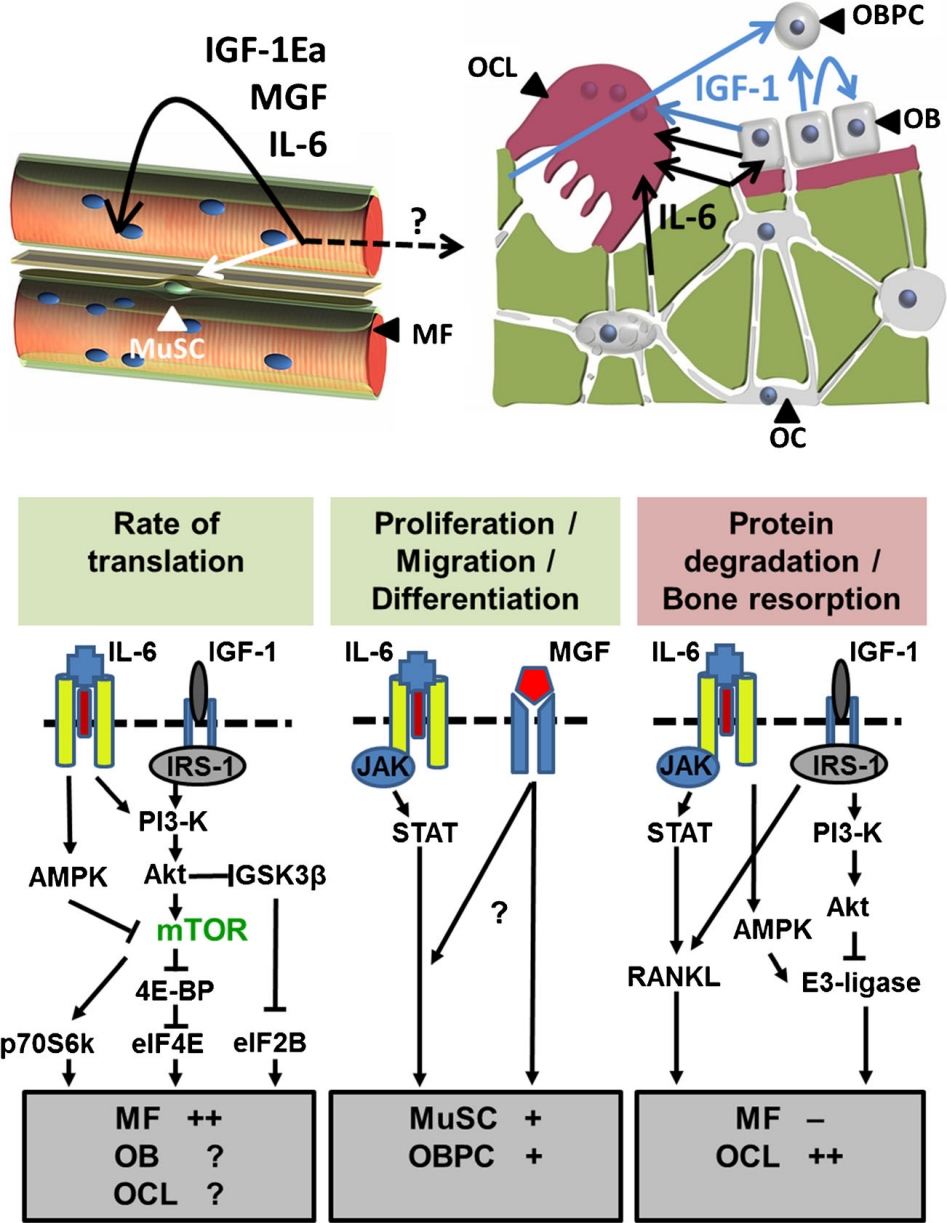
Insulin-like 1 (IGF-1) and interleukin-6 (IL-6) signaling in the regulation of skeletal muscle adaptation and bone metabolism. **a** In skeletal muscle, the machinery for protein synthesis and degradation resides within the muscle fibers, which are multinucleated cells. The muscle satellite cells (MuSCs) reside between the sarcolemma (i.e., plasma membrane) and the basal lamina. **b** In bone, osteoid is synthesized by osteoblasts (OB) which are derived from osteogenic progenitor cells and differentiate into osteocytes (OC) when buried in their own matrix. Osteocytes have long extensions embedded within the canaliculi and are highly sensitive to mechanical loading. In response to mechanical stimuli, myofibers (MF) produce paracrine/endocrine factors such as IL-6, and both splice variants of IGF-1, i.e., mechano growth factor (MGF) and IGF-1Ea. These factors are able to affect myofibers, MuSCs, and cells in other organs such as the liver and potentially bone. IL-6 slows down the rate of translation in myofibers while enhancing the rate of protein breakdown via 5′ adenosine monophosphate-activated protein kinase (AMPK), resulting in a net catabolic effect. On the other hand, IL-6 stimulates the proliferation and differentiation of satellite cells via the Janus kinase/Signal Transducer and Activator of Transcription (JAK/STAT) pathway, which would be an anabolic response. IGF-1 stimulates the rate of protein translation in myofibers via the phosphatidylinositol 3 kinase (PI3-K)/Akt/mammalian target of rapamycin (mTOR) pathway while inhibiting the expression of catabolic ubiquitin E3 ligases resulting in an increase in muscle mass. MGF stimulates satellite cell proliferation and differentiation, but it is so far unclear via which pathway this occurs. Mechanically-loaded osteocytes and osteoblasts produce IGF-1 (likely both splice variants), and IGF-1 is released from the matrix during osteoclastic (OCL) bone resorption. Mechanically-loaded osteocytes and osteoblasts also produce IL-6, as do apoptotic osteocytes. IGF-1 enhances differentiation of osteoblast precursors cells (OBPC) via the PI3-K/AktT/mTOR pathway (not shown) and activity and formation of osteoclasts via stimulation of RANKL expression in osteoblasts and osteocytes. IL-6 also stimulates osteoblast precursor differentiation and osteoclast formation, via an increase in RANKL expression by osteoblasts. Whether IL-6 and IGF-1 affect the rate of protein translation in osteoblasts or osteoclasts is currently unknown. *IRS-1* insulin receptor substrate-1; *eIF4E* eukaryotic initiation factor 4E; *4E-BP* eIF4E-binding protein; *p70S6K* p70S6 kinase; *eIF2B* eukaryotic initiation factor 2B. *GSK3β* glycogen synthase kinase 3β

In skeletal muscle, the application of mechanical loading, either applied passively by stretching or actively by neuronal-initiated contractile activity, stimulates the expression of IGF-1 Ea and MGF [32]. IGF-1 Ea acts on the muscle stem cells (i.e., satellite cells (MuSC) located between the sarcolemma and the basal lamina (Fig. 1) by stimulation of their differentiation into myotubes [32, 33]. In contrast, MGF E peptide has been shown to stimulate proliferation and migration of myoblasts [33, 34]. For an injured muscle fiber, MGF will enhance the capacity for regeneration while for intact mechanically overloaded muscle fibers this will lead to an expansion of the pool of myonuclei within the muscle fiber and as such an increase in the amount of DNA serving as template for transcription. Besides the effects of IGF-1 splice variants on MuSCs, IGF-1 signaling also occurs in the mature muscle fiber. Like insulin, the 70 kD IGF-1 domain stimulates muscle fiber hypertrophy (i.e., increases muscle fiber diameter) [35, 36] by concurrently stimulating the rate of protein synthesis and inhibiting the rate of protein degradation [37]. This dual role makes IGF-1 in muscle an autocrine and paracrine growth factor with a strong potency to induce muscle hypertrophy.

## Interleukin 6

IL-6 is a pleiotropic cytokine which is produced by a variety of cell types such as T-cells and macrophages, but IL-6 is also readily produced by other cell types, such as smooth muscle cells, fibroblasts, skeletal muscle fibers, osteoblasts, and osteocytes [38••, 3]. IL-6 requires gp130 (ubiquitously expressed) and IL-6 receptor (IL-6R) for signaling. IL-6R is expressed by a limited population of cells, amongst which osteoblasts and osteoclasts as well as muscle cells [39]. Human osteocytes express high amounts of IL-6R at week 8 and 14 during gestation, and MLO-Y4 osteocytes have been shown to express IL-6R mRNA [40]. Even if a cell does not express IL-6R, it can respond to IL-6 because IL-6R exists in a soluble form in the serum where it acts as an agonist. IL-6R can end up in the serum because it is shed from cell membranes through cleaving by ADAM17 or ADAM10. Interestingly, osteocytes express ADAM10, making osteocytes possible modulators of IL-6 signaling [41].

IL-6 was first discovered to mediate bone loss associated with estrogen-withdrawal in mice [42]. More recently, it has been described that increased circulating IL-6 levels in patients with Duchenne muscular dystrophy seem responsible for increased osteoclastic bone resorption [43]. IL-6 most likely stimulates osteoclastogenesis indirectly, by increasing Rankl gene expression by osteoblasts [44, 39, 45]. Elevated IL-6 levels could thus explain why the low grade systemic inflammation as occurs after the menopause enhances osteoclastogenesis and reduces bone mass [46]. On the other end of the spectrum, mice *lacking* IL-6 also have a low bone mass [47]. Furthermore, IL-6 null mice show reduced osteoblast numbers and delayed fracture healing [47]. Indeed, IL-6-type cytokines promote differentiation of committed osteoblastic cells toward a more mature phenotype [48], and IL-6 has been shown to enhance osteoblast differentiation in stem cells [49]. This double edged role of IL-6 in bone, i.e., both stimulatory for osteoclasts and osteoblasts, is also reflected in the production of IL-6 by osteocytes. Cultured MLO-Y4 osteocytes produce high amounts of IL-6 [29•]. Fluid shear stress at low physiological levels stimulates IL-6 production in osteocytes in vitro [38••]. On the other hand, IL-6 has also been shown to be expressed by osteocytes when they undergo apoptosis in response to bone unloading [3]. Overall, these data indicate that in bone IL-6 has a key role in bone metabolism.

In skeletal muscle, multiple roles have been identified for IL-6 [50]. Contractile activity stimulates IL-6 expression in humans and rodent skeletal muscle [51, 52], which is paralleled by elevated serum levels in humans [14, 53]. IL-6 produced by muscle is generally known for its role in glycogen metabolism and insulin signaling [54], but IL-6 may also be involved in the regulation of muscle fibers size. Direct chronic IL-6 administration to skeletal muscle induces muscle fiber atrophy [55]. However, recovery of gastrocnemius muscle from disuse atrophy is attenuated in IL-6 knockout mice [56], and overload-induced muscle hypertrophy is blunted in IL-6 deficient mice [57]. These reports suggest opposite roles of IL-6 in the regulation of muscle fiber size and regeneration, similarly to bone.

## Intracellular Signaling Pathways Activated BY IGF-1 AND IL-6

In skeletal muscle, IGF-1 stimulates the rate of protein synthesis via two ways: (1) IGF-1 increases the rate of mRNA transcription of myofilaments such as α-skeletal actin, a major constituent of the muscle contractile apparatus [36, 58], and (2) IGF-1 stimulates the phosphatidylinositol 3 kinase (PI3-K)/Akt (also known as protein kinase B) pathway which activates the mammalian target of rapamycin (mTOR). Downstream targets of mTOR are p70S6 kinase (p70S6k) and heat- and acid-stable protein 1 (PHAS-1, also referred to as 4E-BP) [59]. Activated mTOR phosphorylates P70S6K, thereby activating the ribosomal protein S6, which is involved in the translation of mRNAs [59] (Fig. 1). In addition, mTOR inhibits 4EBP, which is a negative regulator of the translation initiation factor eIF4E, thereby further enhancing the rate of translation [59, 60]. Akt also inactivates glycogen synthase kinase 3 β (GSK3β), thereby preventing the inhibitory effect of GSK3β on eukaryotic initiation factor 2B (eIF2B), which results in an enhanced rate of mRNA translation [61] (Fig. 1). Apart from its potency to enhance the rate of protein synthesis, IGF-1 also attenuates the rate of protein degradation via PI3-K/Akt/FOXO pathway [62]. Activated Akt phosphorylates the transcription factor FOXO, causing its cytoplasmic localization, which reduces the rate of transcription of muscle-specific ubiquitin E3 ligases (Fig. 1). Muscle E3 ligases will tack contractile proteins with ubiquitin, which marks them for degradation within the 26S-proteasome system. Taken together, IGF-1 is a highly potent growth factor resulting in muscle hypertrophy by reducing protein degradation and stimulating the rate of protein synthesis through a combination of increasing mRNA content of contractile proteins and increasing the rate of translation per mRNA.

Although the role of the mTOR pathway in IGF-1-induced muscle hypertrophy has been extensively studied, this pathway attracted little attention in the bone world, until it has been shown recently that IGF-1 may have an anabolic effect on bone via stimulation of the PI3-K/Akt/mTOR pathway [63••]. IGF-1 released from the bone matrix during bone remodeling stimulates osteoblastic differentiation of recruited mesenchymal stem cells (MSCs) by activation of Akt/mTOR [63••]. The observation that mTOR mediates osteoblast differentiation has also been shown in experiments where rapamycin, a potent mTOR inhibitor, suppressed WNT7B-induced osteoblast differentiation in ST2 mouse bone marrow derived cells, as determined by assessing alkaline phosphatase activity and von Kossa staining [64]. As mentioned before, activated Akt not only affects mTOR, but also inhibits GSK3β. Thereby, Akt activates cellular β-catenin signaling in osteocytes [65••], explaining why conditional disruption of IGF-1 in osteocytes abolishes the loading-induced increase in β-catenin protein levels in osteocytes [31••]. The activation of β-catenin by mechanical loading in osteocytes inhibits osteocyte apoptosis [66, 67].

IL-6 signaling occurs through binding of IL-6 with the IL-6 receptors (IL-6R), which subsequently bind to gp130 receptors [68]. In skeletal muscle, both MuScs and myofibers express IL-6, IL-6R, and gp130 receptor. In MuSCs, IL-6-induced proliferation and migration occurs via the JAK/STAT pathway, which stimulates the expression of proliferation-associated transcription factors cyclin D1 and c-myc [57, 69]. Regarding the effects of IL-6 on the myofiber (MF), several studies have shown that IL-6 modulates both the rate of protein synthesis and that of protein breakdown [70–73]. Primary human MuSCs differentiated into myotubes showed increased phosphorylation of Akt after exposure to IL-6 [70, 71] suggesting an enhancement of the mTOR signaling. However, the opposite has been reported in mice overexpressing IL-6 in quadriceps muscle. IL-6 attenuated the activity of mTOR via its stimulatory effect on 5′-adenosine monophosphate-activated protein kinase (AMPK) [72•]. This enzyme is phosphorylated and activated by a low energy status (i.e., increased ratio AMP/ATP), but is also a downstream target of IL-6 [73]. Activated AMPK has a multitude of regulatory functions in skeletal muscle: (1) stimulation of biosynthesis of mitochondria and fatty acid oxidation and glucose uptake [74], (2) inhibition of mTOR [75], and (3) stimulation of expression of muscle-specific E3 ligases [76, 77] which is linearly related to increased muscle protein degradation. The actual effects of IL-6 on the PI3-K/Akt/mTOR pathway within muscle fibers remain to be determined. Note that there may also an indirect way by which IL-6 affect the Akt/mTOR signaling within muscle. Transgenic overexpression of IL-6 or its exogenous injection in mice is associated with reduced circulating IGF-1 levels, likely due to increased proteolysis of the IGF-1 binding protein 3 (IGFBP3) and enhanced clearance of IGF-1 [78].

In bone, IL-6 signals via gp130/IL-6R thereby activating the JAK/STAT pathway, which leads to activation of the transcription factor “nuclear factor kappa-light-chain-enhancer of activated B cells” [79]. IL-6 also activates the Ras/Raf pathway leading to the activation of the transcription factor C/EBPβ, also known as “nuclear factor for IL-6” [80]. The latter binds promoter sequences of amongst others iNOS and COX2 genes and stimulates IGF-1 expression, which all play a role in the regulation of bone metabolism [81]. The C/EBPβ gene gives rise to three proteins: LAP*, LAP, and LIP. LAP mediates osteoblast maturation and osteoblast-stimulated osteoclastogenesis while LIP inhibits osteoblast-stimulated osteoclastogenesis [82]. In osteoclasts, the balance between LAP and LIP expression is determined by mTOR activity, i.e., low mTOR activity favoring LAP, while high mTOR activity favors LIP [83]. Whether IL-6 affects mTOR activity in osteoblasts and/or osteocytes is currently unknown.

## Role for the mTOR Pathway in Bone

From the foregoing, it is clear that there are similarities and dissimilarities in the way IGF-1 and IL-6 are employed by bone and muscle to achieve changes in tissue mass, and that much can be learned from one field in order to generate a better understanding of regulation of tissue mass in the other field. A similarity between muscle and bone is that the balance between protein formation and degradation determines tissue mass. In order for protein formation to increase in muscle, the muscle increases the amount of nuclei (DNA) per fiber, the amount of RNA per available myonucleus (rate of transcription), as well as the rate of translation per mRNA molecule. In bone, the protein matrix is deposited outside of the cells rather than intracellular, and bone formation and degradation occur by two separate cell types, but otherwise it makes sense that in order for bone matrix production to increase, the same principles apply as for muscle. Indeed, an increase in proliferation and differentiation of osteoblast precursors increases the number of osteoblast nuclei (DNA) per volume bone. For example, MGF enhances the number of osteogenic cells in bone through stimulation of proliferation [23]. One might then question: what is next? In order to increase bone formation rate in an efficient manner, the activity per osteoblast needs to be enhanced as well. The master switch in the rate of protein translation in muscle, as in many other cell types, is the PI3-K/Akt/mTOR pathway, as discussed above [84]. It is possible that mTOR plays a very analogous role in osteoblasts, although the little evidence that is currently available for such a role is ambiguous. On the one hand, genetic disruption of mTOR signaling by deleting Raptor for 3 weeks in the osteoblast lineage in 1-month-old mice did not affect bone mass or strength, suggesting that mTOR signaling in osteoblasts may not be essential for maintaining bone homeostasis on the short term [64]. On the other hand, targeted induction of the WNT7B gene in osteoblasts dramatically enhanced bone mass due to increased osteoblast number, activity, and significantly stimulated bone formation, but not when the cofactor for mTOR signaling Raptor was absent [64]. Thus, Wnt-7B requires mTOR to promote bone formation [64]. In addition, the importance of mTOR signaling for osteoblasts is exemplified by the experiments in which PI3K/mTOR inhibitors, designed as oncostatic drugs, were tested in vivo. Male mice received amongst others the PI3-K/mTOR inhibitors EZ235 and PI103, which reduced BV/TV by 36 and 37 %, respectively [85••]. Of course, this could still be through an effect of mTOR on osteoblast differentiation rather than on the rate of translation.

Whether the mTOR pathway is important for osteocytes is unknown, but it stands to reason that this pathway affects osteocyte biology. When oxygen and nutrients are not abundant, for instance in osteocytes that are not in direct contact with the vasculature, it makes sense that these cells slow down their metabolism and recycle cell components as much as possible through autophagy. Since mTOR activation enhances the energy consuming process of protein translation and inhibits autophagy [86•], one could deduce that mTOR activity needs to stay low in unstimulated osteocytes [87]. Alterations in mTOR activity could thus have profound effects on osteocyte survival and bone metabolism.

## Conclusion

Taken together, IL-6 and IGF-1 are extremely important regulators of bone and muscle metabolism, which makes them interesting targets for the treatment of osteopenia or sarcopenia. However, IGF-1 and IL-6 are ubiquitously expressed and signal in many cell types, which makes it difficult to target these molecules only in bone and muscle but not in other tissues. The same holds for the signaling pathway that IGF-1 and IL-6 have in common, i.e., the mTOR pathway. As the mTOR pathway is ubiquitously involved in many cells types, activation of this pathway specifically in bone will be a challenge, although targeted delivery of chemicals using bisphosphonates may be an option. However, even if bone can be reached as a single target, IGF-1, IL-6, and mTOR signaling seem to be a two-edged sword: All seem to stimulate osteogenic differentiation and might be essential in bone development and healing. Knockout of IL-6 in otherwise healthy animals and inhibition of mTOR with compounds such as rapamycin certainly have deleterious effects in bone mass. However, both IGF-1 and IL-6 may also stimulate bone resorption, and mTOR activation in osteoclast precursors stimulates osteoclast formation [83]. IL-6 inhibitors are available in the clinic for suppression of inflammation in rheumatoid arthritis, where they seem to be osteoanabolic, rather than catabolic [88]. Pharmacological intervention in these pathways will thus require fine tuning of dose and kinetics (e.g., continuous or intermitted) in order to achieve an adequate anabolic effect.

Alternatively, one could try to specifically target IL-6 and IGF-1 signaling in muscle by pharmacological means, in order to increase both muscle and bone mass. Indeed, overexpression of IGF-1 in the musculature of mice enhanced bone mass as well [89]. Increased muscle mass could be anabolic for bone since muscles exert mechanical forces in bone. Exercise, which stimulates IL-6 and IGF-1 production in bone and muscle, as well as the production of a large quantity of other growth factors and signaling molecules, may be a safe and efficient way to enhance both bone and muscle mass.

In muscle, the mTOR pathway is activated via the food supplement leucine, which transiently stimulated the rate of translation in muscle cells in vitro [90, 91]. Whether supplements can be applied to affect the proliferation of osteoblasts and the rate of translation in these cells is yet unknown and remains to be determined. However, until very fine tuned and tissue-specific pharmacological interventions are available, a combination of sufficient exercise and a balanced diet containing sufficient amounts of for example leucine are a safe and cheap way to help maintain muscle and bone mass.
